# Bufalin suppresses hepatocellular carcinoma invasion and metastasis by targeting HIF-1α via the PI3K/AKT/mTOR pathway

**DOI:** 10.18632/oncotarget.7935

**Published:** 2016-03-06

**Authors:** Haiyong Wang, Chenyue Zhang, Litao Xu, Kun Zang, Zhouyu Ning, Feng Jiang, Huiying Chi, Xiaoyan Zhu, Zhiqiang Meng

**Affiliations:** ^1^ Department of Integrative Oncology, Fudan University Shanghai Cancer Center, Shanghai 200032, China; ^2^ Department of Radiation Oncology, Shandong Cancer Hospital & Institute, Jinan, 250117, China; ^3^ Department of Oncology, Shanghai Medical College, Fudan University, Shanghai 200032, China; ^4^ Department of Integrative Oncology, Zhucheng Hospital of TCM, Zhucheng 262200, China; ^5^ Shanghai Geriatric Institute of Chinese Medicine, Longhua Hospital, Shanghai University of Traditional Chinese Medicine, Shanghai 200031, China

**Keywords:** bufalin, metastasis, HIF-1α, hepatocellular carcinoma, PI3K/AKT/mTOR

## Abstract

It has been reported that there are multiple mechanisms by which bufalin could exert its antimetastatic effect. HIF-1α has been reported to be involved in tumor migration and invasion by regulating EMT. However, it is not known whether bufalin could exert the antimetastatic effect by modulating HIF-1α expression in hepatocellular carcinoma. In the present study, we aimed to evaluate the antimetastatic potential of bufalin *in vivo* and *in vitro*. Our results demonstrated that the liver/lung metastases were significantly reduced in bufalin-treated mice, as tested in the orthotopic transplanted and tail vein injection tumor models. Furthermore, the epithelial-to-mesenchymal transition (EMT) was inhibited in bufalin-treated tumors, as reflected the upregulation of E-cadherin, and downregulation of N-cadherin, vimentin, Snail. Similar results were observed in SMMC7721 cells treated with bufalin. Moreover, the transforming growth factor-β1 (TGF-β1)-induced EMT was also abrogated by bufalin. Mechanistically, our study demonstrated that hypoxia-inducible factor-1α (HIF-1α) played an important role in the antimetastatic effect of bufalin in hepatocellular carcinoma. Importantly, HIF-1α expression may be regulated through the inhibition of the PI3K/AKT/mTOR pathway. Taken together, our results suggest that bufalin suppresses hepatic tumor invasion and metastasis and that this process may be related to the PI3K/AKT/mTOR/ HIF-1α axis.

## INTRODUCTION

Hepatocellular carcinoma (HCC) is the fifth most common malignancy worldwide and the second leading cause of cancer-related deaths in Asia, particularly in China [1]. Currently, surgical resection and liver transplantation are the best options for treating HCC [2–4]. However, most HCC patients are diagnosed at an advanced stage, thus losing such opportunities. Recurrence and metastasis are the major obstacles to improved survival for HCC patients [5]. Therefore, the inhibition of tumor metastasis is of vital importance in clinical practice.

Some studies have suggested that bufalin exhibits significant anti-tumor activity via the inhibition of cell proliferation and angiogenesis, induction of apoptosis, et al. [6]. However, studies reporting bufalin's antimetastatic effect are relatively limited. Recently, studies reported that bufalin can inhibit migration and invasion by modulating TJs and MMPs in T24 cells [7]. Emerging evidence suggests that the epithelial-to-mesenchymal transition (EMT) is a key process in tumor invasion and metastasis. During this process, epithelial cells lose their epithelial properties, while acquiring mesenchymal properties that include morphology, cellular structure, and biological function [8]. These changes enhance tumor cell migration and allow tumor cells to metastasize and establish secondary tumors at distant sites [9–10]. Importantly, EMT has also been reported to be involved in HCC progression and correlates with patient prognosis [11].

VEGF functions as a primary stimulus for angiogenesis. VEGF binds to VEGF receptor, which leads to the activation of phosphatidylinositol 3-kinase (PI3K)/AKT signaling pathway. PI3K/AKT signaling regulates angiogenesis through affecting the expression of VEGF [12]. A hypoxic environment causes HCC cells to activate the hypoxia response that subsequently leads to pro-survival reactions, elevated angiogenesis, adapted metabolic alterations, tumor invasion and metastasis [13]. Hypoxia-inducible factor-1α (HIF-1α) is a critical mediator of the physiological response to hypoxia, and its dysregulation can promote tumor angiogenesis and metastasis [14].

It has also been documented that HIF-1α is required to transcriptionally regulate VEGF expression. For instance, Y-H2AX has been reported to promote HCC angiogenesis via EGFR/HIF-1α/VEGF pathways under hypoxic condition [15]. And HIF-1α/GPER/VEGF signaling in cancer cells was also activated by copper [16]. Besides being a VEGF modulator, HIF-1α may be a master regulator of EMT, as some studies have been suggested [17-18]. Importantly, HIF-1α has been shown to promote HCC invasion and metastasis by inducing EMT. The possible molecular mechanism is that HIF-1α upregulates Snail expression to indirectly affect the levels of E-cadherin, N-cadherin, and vimentin [19]. Recently, studies have shown that the upregulation of HIF-1α transcription and stabilization, which subsequently induce an EMT response in the HCC cells, is significantly associated with increased metastatic activity in HCC [20].

Studies have shown that PI3K/AKT can regulate HIF-1α expression and is also involved in EMT [19, 21]. However, it is unclear whether bufalin can affect EMT by targeting HIF-1α via modulating PI3K/AKT pathway.

Therefore, in the present study, we evaluated bufalin's effect on HCC migration and invasion, and tested whether this effect was linked with PI3K/AKT/ HIF-1α pathway.

## RESULTS

### Bufalin inhibits hepatic tumor metastasis *in vivo*

**Figure 1:**
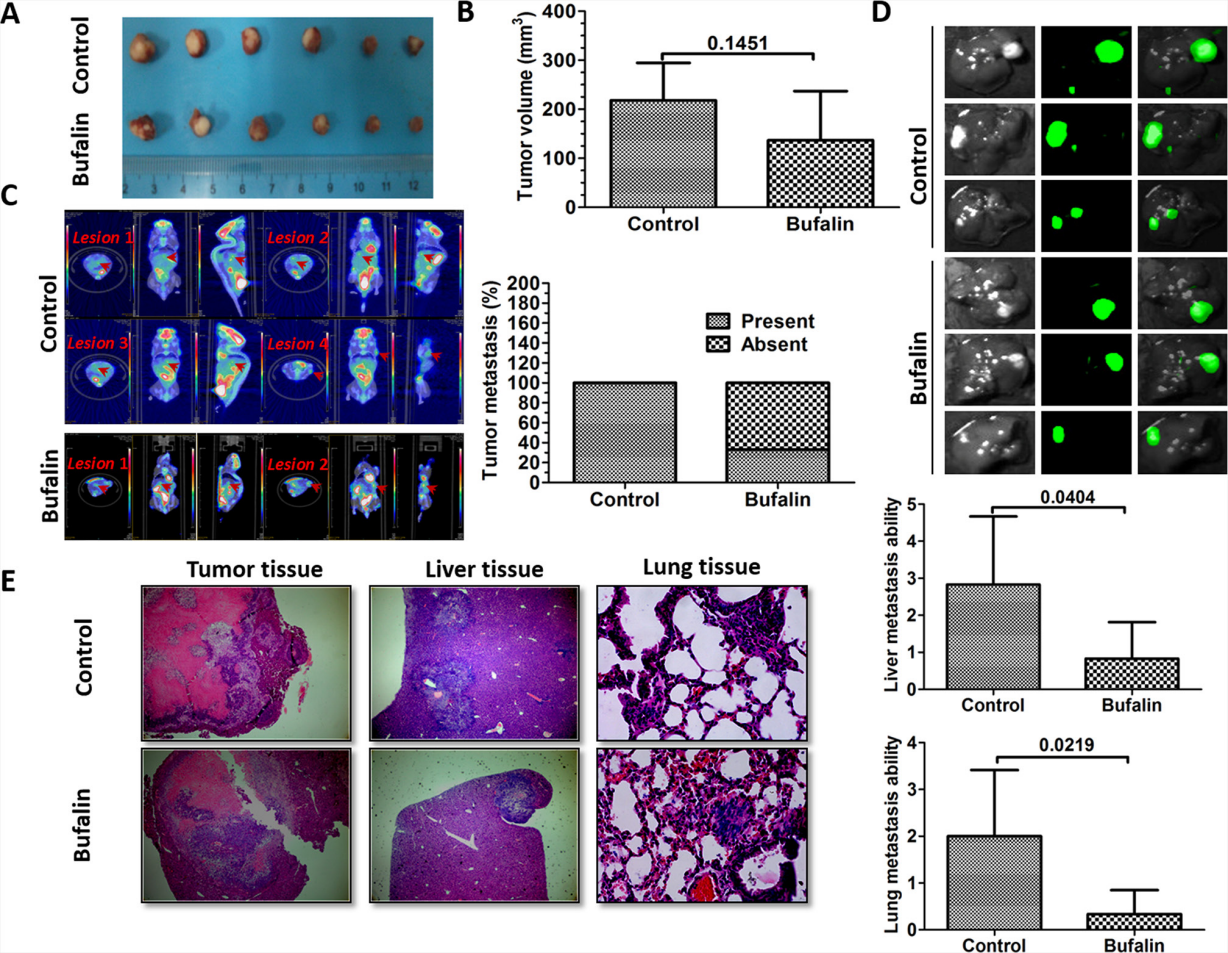
Bufalin inhibits the metastasis of hepatic tumors *in vivo* The mice were orthotopically implanted with the SMMC7721-GFP cells and treated with bufalin (1 mg/kg, 5 days/week) via an intraperitoneal injection, for six weeks. (**A**) Images of orthotopic-transplanted tumors from both the groups are shown. (**B**) The bar graph shows the volume of the hepatic tumors. At the end of the experiment, the primary tumors from the control and the bufalin-treated mice were carefully excised, and their volumes were measured. (**C**) Representative images captured by the Inveon micro PET/CT system to visualize metastasis in the animals from the control and bufalin-treated groups (the representative mouse in the control group has four metastatic lesions, and the mouse in the bufalin-treated group has two metastatic lesions; the red arrows represent tumor lesions). The bar graph shows the percentage of tumor metastases in each group. (**D**) Representative images captured by the Lumazone imaging system to visualize intrahepatic metastasis in animals from the control and the bufalin-treated groups. (**E**) Tumor tissue, liver tissue and lung tissue were analyzed using HE staining (40× for tumor and live tissue, 400× for lung tissue). Liver and lung metastases were quantified by counting the number of metastatic colonies in one histological section of the mid-portion of each liver or lung sample from each mouse. Representative images and dot plots are shown. The data are expressed as the mean ± SD.

In the present study, we investigated whether bufalin could inhibit HCC metastasis. Our results showed that bufalin slightly reduced primary tumor growth compared with the control tumors (Figure 1A and 1B). However, fewer metastatic lesions were detected in bufalin-treated mice, as shown using the Inveon micro PET/CT system. In fact, two out of the three mice in the treated group showed no metastatic lesions, whereas in the control group, all three mice (100%) had metastatic lesions (Figure 1C). Intrahepatic metastatic lesions were further detected using the Lumazone imaging system. Fewer intrahepatic metastatic nodules were detected in bufalin-treated mice (six mice in each group) (Figure 1D). Tumor, liver and lung tissues were further analyzed by hematoxylin-eosin (HE) staining and observed under a microscope (40× for tumor and live tissue, 400× for lung tissue). Interestingly, tumor nodule infiltration was observed at the edge of the tumor tissue in the control mice, and an increased number of liver and lung metastatic nodules was also detected in the control mice (*P* = 0.040, *P* = 0.0219) (Figure 1E).

### Bufalin inhibits systemic metastasis of SMMC7721-GFP cells *in vivo*

**Figure 2:**
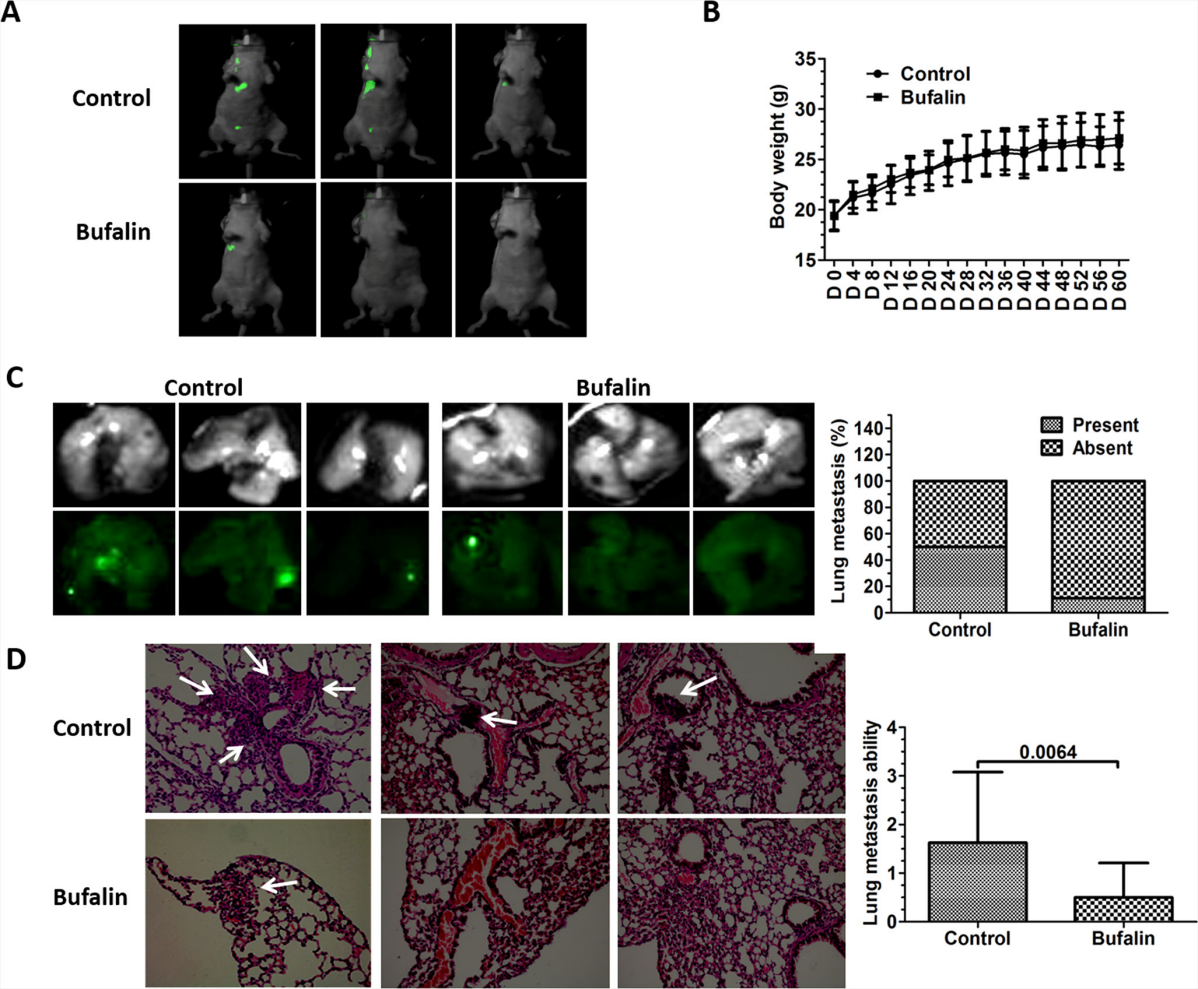
Bufalin inhibits the systemic metastasis of SMMC7721-GFP cells *in vivo* The systemic metastasis model was established by injecting the mice with SMMC7721-GFP cells via the tail vein. The mice received intraperitoneal injections of bufalin (1 mg/kg, 5 days/week) for 8 weeks. (**A**) Representative images of mice from the control and bufalin-treated groups captured using the Lumazone imaging system to visualize metastasis. (**B**) The bar graph shows no difference in body weight between the control and the bufalin-treated mice. The data are expressed as the mean ± SD. (**C**) The representative images of the lung tissues from the control and bufalin-treated mice using the Lumazone imaging system to visualize metastasis. The percentage of the lung metastases in the control and the bufalin-treated mice are summarized in a bar graph. (**D**) The representative images showing HE staining (200×) that was performed to detect any lung metastasis in the control and the bufalin-treated mice (the white arrows represent micro metastases). A bar graph is shown to summarize the lung metastases in each group. The lung metastases were quantified by counting the number of metastatic colonies in one histological section of the mid-portion of each lung sample from each mouse. The data are expressed as the mean ± SD.

In an orthotopic metastasis model, we demonstrated that bufalin inhibited HCC metastasis. To further confirm the inhibitory potential of bufalin on tumor metastasis, mice injected with SMMC7721-GFP cells via their tail veins were treated with 1 mg/kg bufalin (5 days/week) through an intraperitoneal injection, and systemic metastases were detected using the Lumazone imaging system. Our results showed that bufalin-treated mice developed fewer metastatic lesions, and fewer mice were detected with metastasis in the bufalin-treated group compared with those in the control group (Figure 2A). Importantly, fewer mice had lung metastases in the bufalin-treated group compared with those in the control group (Figure 2C). The presence of lung metastases was further analyzed by HE staining, under a microscope (200×). As predicted, fewer lung metastatic nodules were detected in the bufalin-treated group (*P* = 0.0064). Interestingly, tumor-like thrombi were detected in the control mice (Figure 2D). Additionally, the bufalin treatment was well tolerated by the mice, as indicated by no weight loss or signs of acute or delayed toxicity (Figure 2B).

### Bufalin inhibits EMT *in vivo*

**Figure 3:**
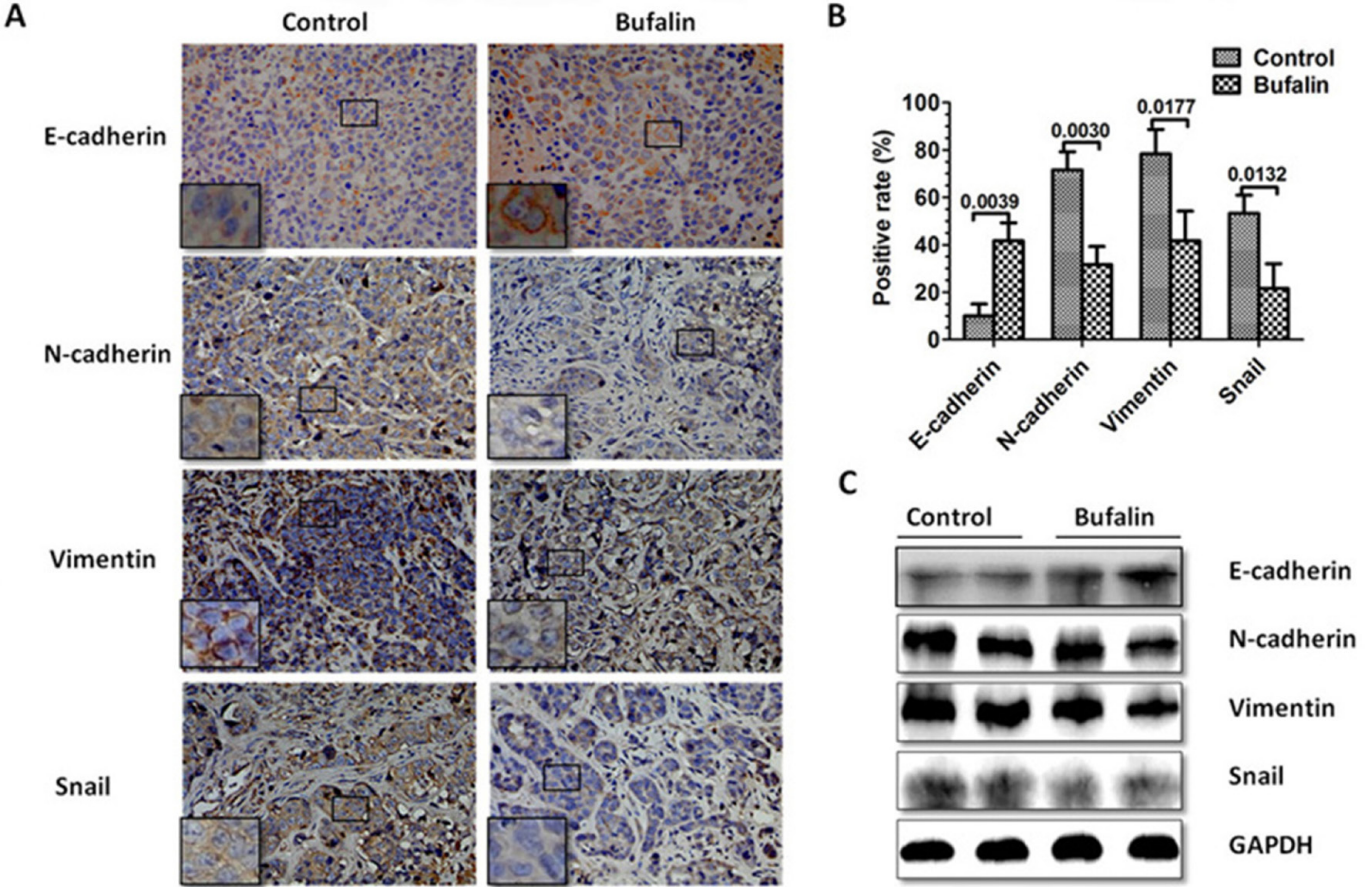
Bufalin inhibits the EMT *in vivo* At the end of the experiment in an orthotopic xenograft model, the control and the bufalin-treated tumors were excised and analyzed using immunohistochemistry and western blot to measure the expression of EMT markers. (**A**) IHC staining with anti-E-cadherin, anti-N-cadherin, anti-vimentin, and anti-Snail antibodies were performed using sections of the orthotopic transplanted tumors. (**B**) The positive rate of the EMT-related markers E-cadherin, N-cadherin, vimentin and Snail are based on IHC. (**C**) Western blot analysis was used to measure the expression of the EMT-related markers E-cadherin, N-cadherin, vimentin and Snail in the cells derived from two representative xenograft samples from each group. GAPDH was used as a loading control in the western blot analysis.

EMT closely correlates with cancer metastasis [7–8]. The inhibition of hepatic tumor metastases by bufalin *in vivo* prompted us to examine whether the antimetastatic effect of bufalin was due to the inhibition of the EMT. Immunohistochemical and western blot analyses were used to investigate the effect of bufalin on EMT. Marginal tissues of the xenografts were removed, and immunohistochemical staining was applied. The expression of EMT-related markers was then evaluated. The epithelial marker E-cadherin was upregulated, and the mesenchymal markers N-cadherin, Vimentin, and Snail were downregulated in the bufalin-treated mice compared with the control group (Figure 3). These data suggested that bufalin could inhibit EMT in human HCC.

### Bufalin inhibits TGF-β1-induced EMT in SMMC7721 cells

**Figure 4:**
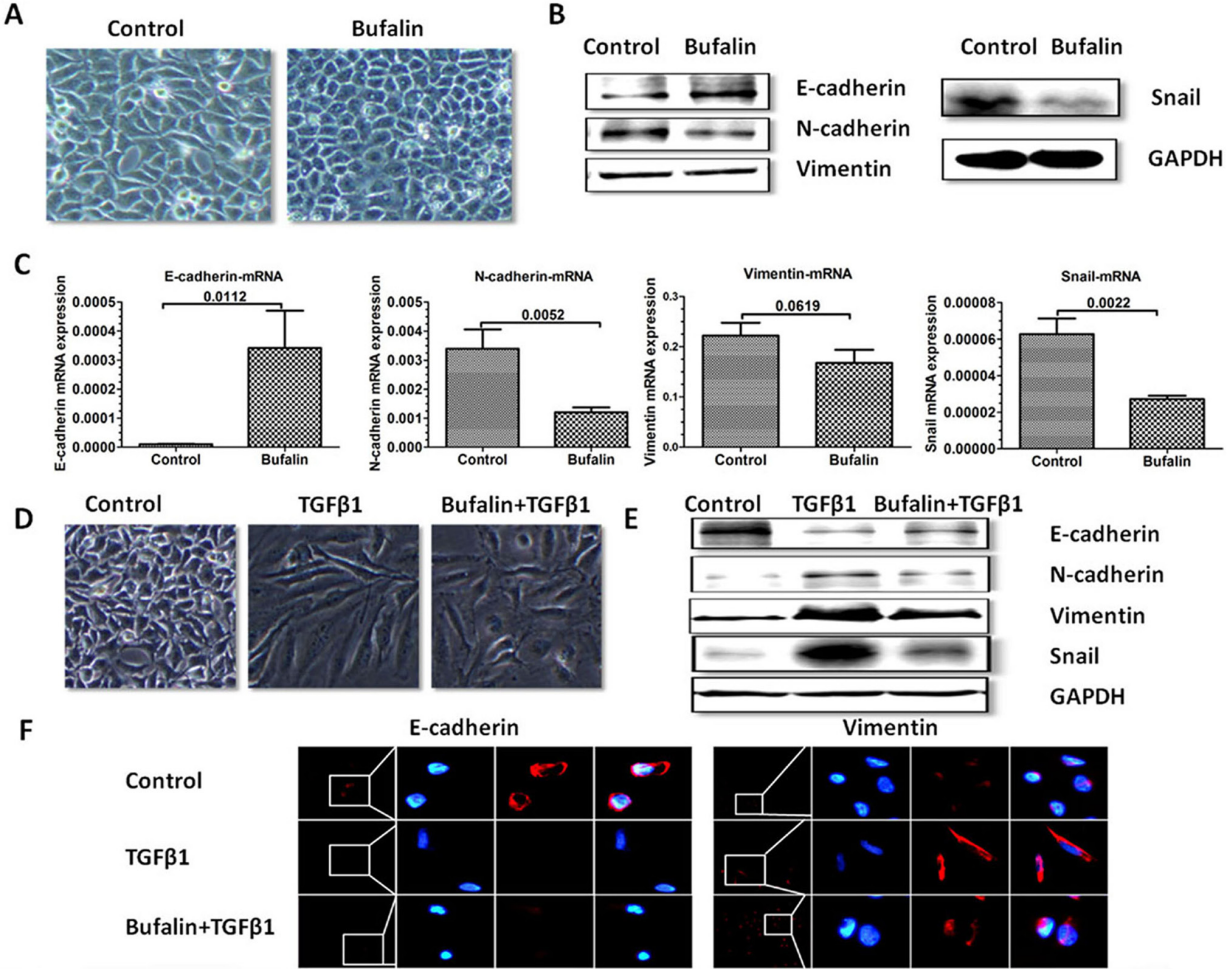
Bufalin inhibits TGF-β1-induced EMT in SMMC7721 cells The SMMC7721 cells were serum starved and treated with TGF-β1 or bufalin, or both, and the expression of the EMT markers was measured. (**A**) After 24 h of treatment with bufalin (10 nM), the morphological changes of the SMMC7721 cells were evaluated. (**B** and **C**) The expression of the EMT markers E-cadherin, N-cadherin, vimentin and Snail in the SMMC7721 cells was assessed by western blot analysis and RT-PCR. GAPDH was used as a loading control in the western blot analysis. Bar graph summarize the expression levels of the EMT markers. (**D**) After 72 h of combined treatment with bufalin (5 nM) and TGF-β1 (10 ng/ml), the morphological changes in the SMMC7721 were evaluated. (**E** and **F**) The expression of E-cadherin, N-cadherin, vimentin and Snail was assessed by western blot analysis and immunofluorescence. GAPDH was used as a loading control in the western blot analysis.

Having shown that bufalin inhibited the primary tumor growth and the metastases of the hepatic tumors by inhibiting EMT *in vivo*, we next investigated whether bufalin could inhibit the EMT of hepatic cancer cells *in vitro*. We examined the expressions of EMT markers in both bufalin-treated and control hepatoma cells. As expected, the morphology of the SMMC7721 cells treated with 10 nM bufalin tended to change into a ‘square’ epithelial-like appearance after 24 h (Figure 4A). The expression of EMT markers was then measured by western blot analysis and RT-PCR. The results showed an upregulation of the epithelial marker E-cadherin and a downregulation of the mesenchymal markers N-cadherin, vimentin and Snail in bufalin-treated SMMC7721 cells (Figure 4B and 4C). Members of the TGF-β family of growth factors can initiate and maintain the EMT in a variety of biological systems, and TGF-β1 is a major inducer of the EMT in cancer [18–19]. In the present study, SMMC7721 cells treated with 10ng/ml TGF-β1 showed an elongated morphology. However, bufalin treatment substantially reduced the TGF-β1-mediated changes in SMMC7721 cell morphology (Figure 4D). Importantly, western blot and immunofluorescence revealed that TGF-β1 induced the downregulation of the epithelial marker E-cadherin, while the upregulation of the mesenchymal markers N-cadherin, vimentin and Snail was inhibited in the bufalin-treated group (Figure 4E and 4F).

### Bufalin inhibits TGF-β1-induced invasion and migration of SMMC7721 cells

**Figure 5:**
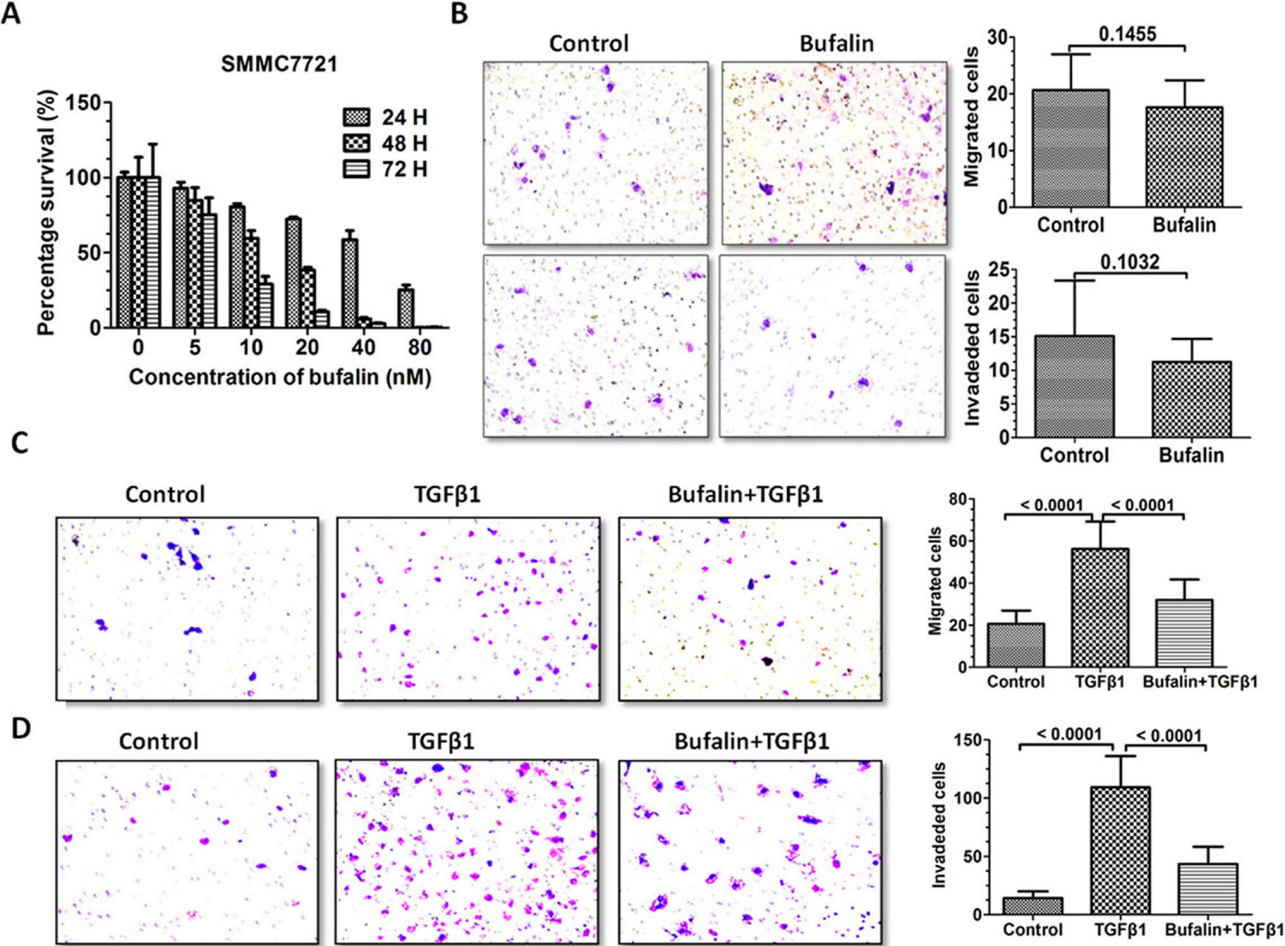
Bufalin inhibits TGF-β1-induced invasion and migration of SMMC7721 cells (**A**) The cells were plated in a 96-well plate and treated with various concentrations of bufalin; viability was measured using a Cell Counting Kit-8. (**B**) The migration and invasion of the SMMC7721 cells was measured using the transwell assay. (**C**) SMMC7721 cell migration was measured using the transwell assay after TGF-β1 treatment. The data are expressed as the mean ± SD. (**D**) SMMC7721 cell invasion was also measured using the transwell assay after TGF-β1 treatment. The data are expressed as the mean ± SD.

Having shown that bufalin inhibited TGF-β1-mediated EMT in SMMC7721 cells, we next examined whether the inhibitory effect of bufalin on EMT could inhibit SMMC7721 cell invasion and migration. A cell proliferation assay was applied to measure the effects of the bufalin treatment on the proliferative capability of the hepatoma cells. We observed an anti-proliferative effect of bufalin on SMMC7721 cells, which was dose and time dependent at a concentration of 5–80 nM (Figure 5A). However, due to the low metastatic potential of SMMC7721 cells, SMMC7721 cell invasion and migration were not significantly reduced in the bufalin-treated group (Figure 5B). Interestingly, the TGF-β1-induced increase in SMMC7721 cell migration and invasion was significantly reversed by bufalin (*P* < 0.0001) (Figure 5C and 5D).

### Bufalin inhibits HIF-1α expression

**Figure 6:**
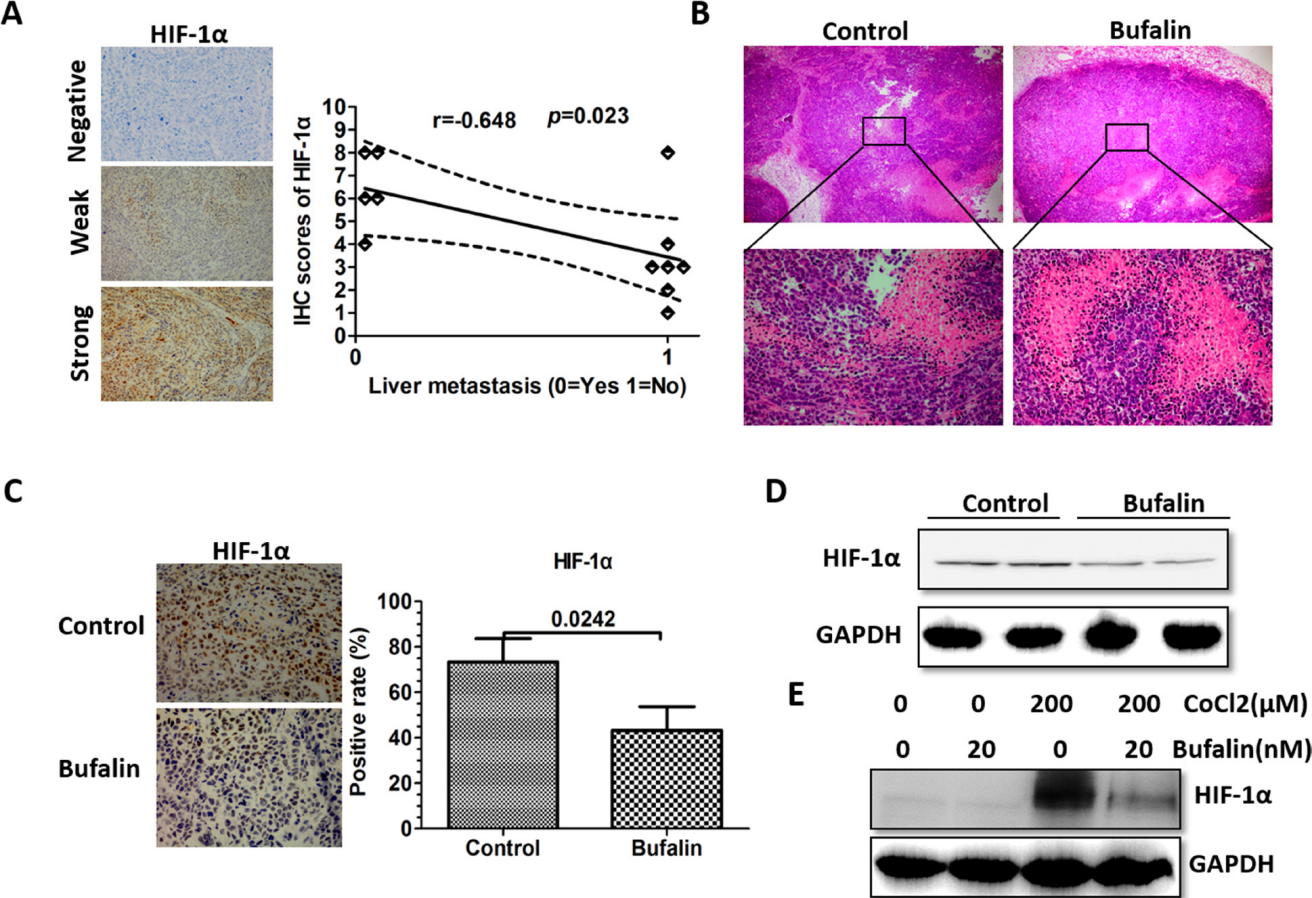
Bufalin inhibits HIF-1α expression (**A**) The correlation analysis between the IHC scores of HIF-1α and the intrahepatic metastasis in the orthotopic transplanted tumors. (**B**) H & E staining was performed to measure necrosis. (**C**) IHC staining with anti-HIF-1α was performed using sections of the orthotopic transplanted tumors, and the positive rate of the HIF-1α was based on IHC. (**D**) Western blot analysis was used to measure HIF-1α expression in cells derived from two representative xenograft samples from each group. GAPDH was used as a loading control in the western blot analysis. (**E**) After 24 h of treatment with CoCl2 (200 μ M), HIF-1α expression was assessed using western blot analysis. GAPDH was used as a loading control.

HIF-1α is a signal transcription factor that plays an important role in many critical aspects of HCC tumorigenesis, progression, and metastasis [14]. Previous research has demonstrated that HIF-1α promotes HCC invasion and metastasis by inducing EMT [19]. Consistent with that study, the orthotopic xenograft tissues that produce liver metastases also showed increased HIF-1α expression, as determined by IHC (Figure 6A). Therefore, immunohistochemical and western blot analyses were used to investigate the effect of bufalin on HIF-1α. As expected, HIF-1α expression was downregulated in bufalin-treated mice, and there was an increase in necrosis (Figure 6B–6D). In addition, CoCl_2_, a hypoxia-inducing agent, increased HIF-1α expression. Interestingly, the CoCl_2_-induced HIF-1α expression was abrogated by bufalin (Figure 6E).

### HIF-1α mediated EMT and VEGF involved in the antimetastatic process of bufalin

**Figure 7:**
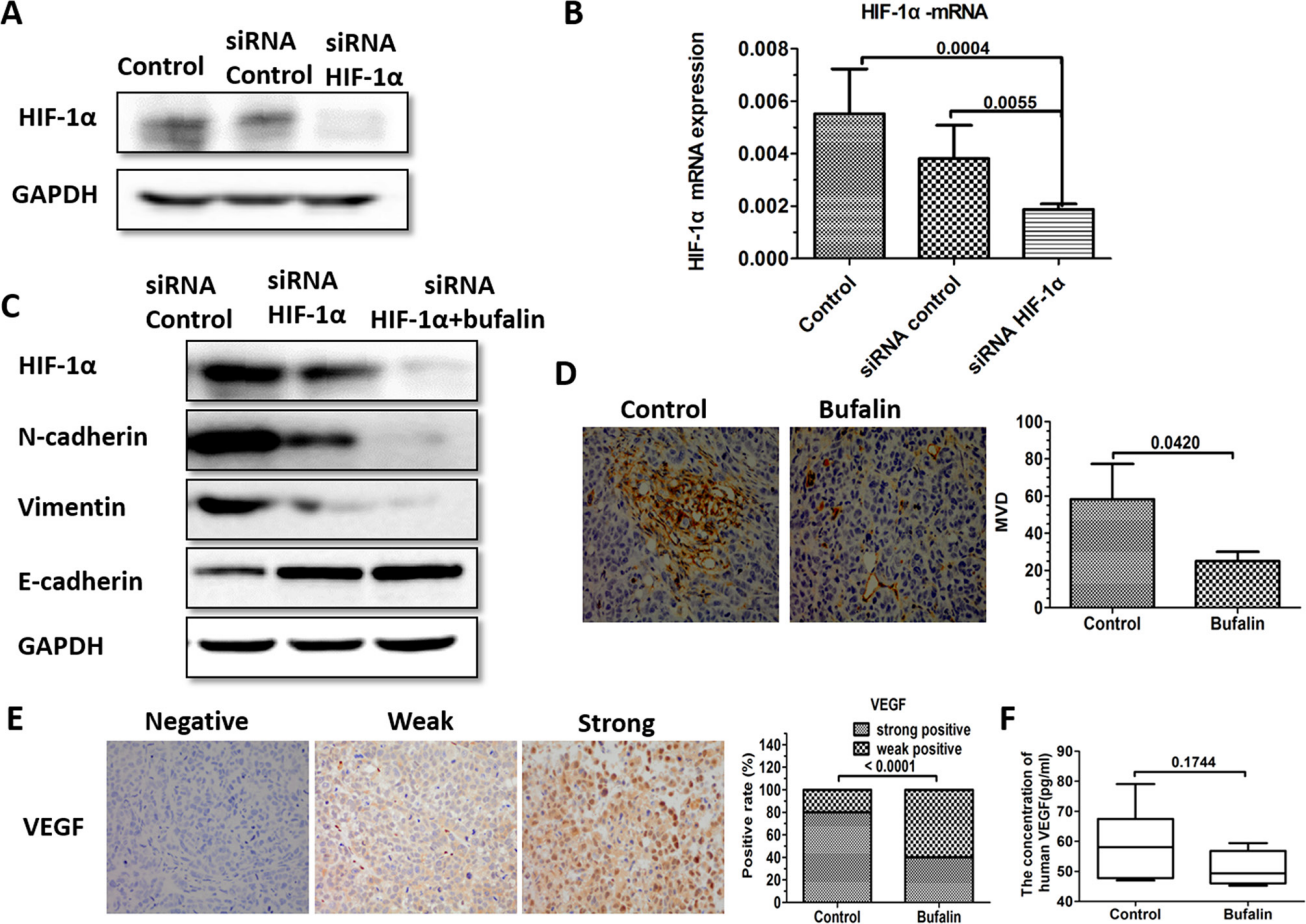
HIF-1α mediated EMT and VEGF involved in the antimetastatic process of bufalin (**A**) The HIF-1α protein expression in SMMC7721 HCC cell lines transfected with Control siRNA and HIF-1α siRNA. (**B**) The HIF-1α mRNA expression in SMMC7721 HCC cell lines transfected with Control siRNA and HIF-1α siRNA. (**C**) HIF-1α, E-cadherin, Vimentin and N-cadherin were detected in SMMC7721 cells treated with different treatments by Western blot (Control siRNA, HIF-1α siRNA, and bufalin+ HIF-1α siRNA) (**D**) IHC staining with anti-CD31 was performed using sections of the orthotopic transplanted tumors to assess MVD. Bar graphs summarize the MVD in each group. The data are expressed as the mean ± SD. (**E**) IHC staining with anti-VEGF was performed using sections of the orthotopic transplanted tumors and the positive rate of VEGF based on IHC. (**F**) The concentration of human VEGF in the serum in each mouse.

Having shown that bufalin inhibits the process of EMT and the expression of HIF-1α, we employed the RNA interference for HIF-1α for 48 h and 72 h, respectively. The mRNA and protein expressions of were detected in SMMC7721 cell. As reflected in the real time PCR and western blot, the mRNA and protein levels were both downregulated in SMMC7721 cells transfected with siRNA targeting HIF-1α as compared to the ones transfected with nontarget siRNA (Figure 7A–7B). As downregulation of HIF-1α significantly can reverse EMT, we detected crucial proteins such as E-cadherin, N-cadherin, Snail and Vimentin in EMT. Since we have shown that HIF-1α may be one of the targets of bufalin, EMT-related proteins were also detected in SMMC7721 cells treated with both bufalin and siRNA targeting HIF-1α. Results have shown that the suppression of EMT was more evident in SMMC7721 cells treated with both bufalin and HIF-1α siRNA (Figure 7C). In addition, we have found that buflain could downregulated HIF-1α and increased necrosis in tumor tissues, we hypothesize that microvessels may be inhibited by bufalin. As expected, the microvessel density (MVD) was inhibited in the bufalin-treated group (*P* = 0.0420) (Figure 7D). Additionally, HIF-1α could mediate the expression of VEGF, an important factor that promotes angiogenesis and is involved in tumor angiogenesis [22]. Next, immunohistochemical analysis revealed that bufalin suppressed VEGF expression (Figure 7E). ELISA analysis further revealed that VEGF was downregulated in mouse serum in the bufalin-treated group (Figure 7F).

### Bufalin inhibits the PI3K/Akt/mTOR signaling pathway

**Figure 8:**
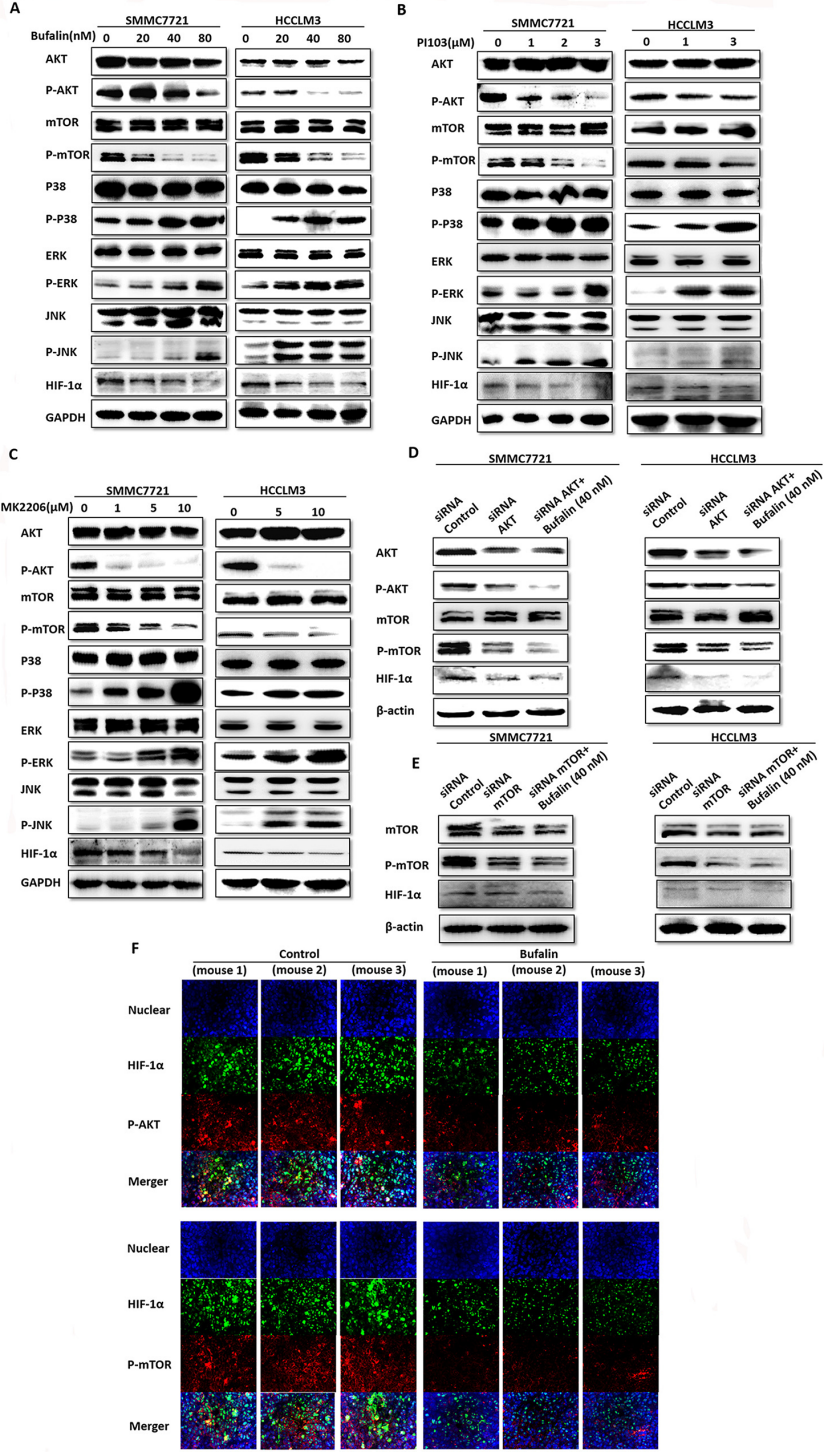
Bufalin inhibits the PI3K/Akt/mTOR/HIF-1α signaling pathway (**A**) SMMC7721 and HCCLM3 cells were treated with bufalin in a dose- and time-dependent manner. The PI3K/Akt and Ras/MAPK pathways and HIF-1α were assessed by western blot analysis. GAPDH was used as a loading control. (**B** and **C**) SMMC7721 and HCCLM3 cells were treated with different doses of PI103 and MK2206. The PI3K/Akt and Ras/MAPK pathways and HIF-1α were assessed by western blot analysis. GAPDH was used as a loading control. (**D** and **E**) SMMC7721 and HCCLM3 cells were treated with siRNA-AKT and siRNA-mTOR. The PI3K/Akt/mTOR pathways and HIF-1α were assessed by western blot analysis. GAPDH was used as a loading control. (**F**) The expression of p-AKT, p-mTOR, HIF-1α were assessed by immunofluorescence.

The PI3K/AKT and Ras/MAPK pathways are involved in the regulation of HIF-1α [23]. Western blot analysis was used to investigate the effect of bufalin on the two pathways. Surprisingly, the PI3K/AKT/mTOR pathway was inhibited, and the Ras/MAPK pathway was activated by bufalin, in a dose- and time-dependent manner along with the inhibition of HIF-1α expression (Figure 8A). We then used PI103 and MK2206 to inhibit the PI3K/AKT/mTOR signaling pathways. Interestingly, HIF-1α expression was also inhibited in a dose-dependent manner and the Ras/MAPK pathway was activated (Figure 8B–8C). Next, we used siRNA-AKT and siRNA-mTOR to inhibit the PI3K/AKT/mTOR pathway. As expected, HIF-1α expression was also inhibited (Figure 8D–8E). To further validate our conclusion, immunofluorescence was used to observe the expression of PI3K/AKT/mTOR signaling pathways and HIF-1α, we found p-AKT, p-mTOR and HIF-1α expression were also downregulated in bufalin-treated mice (Figure 8F). Our results showed that the PI3K/AKT/mTOR pathway plays a main role in mediating HIF-1α expression. The activation of the Ras/MAPK pathway may be a protective mechanism to adapt to the endoplasmic reticulum (ER) stress [24].

### Diagram of the proposed mechanism by which bufalin inhibits hepatocellular carcinoma invasion and metastasis

**Figure 9:**
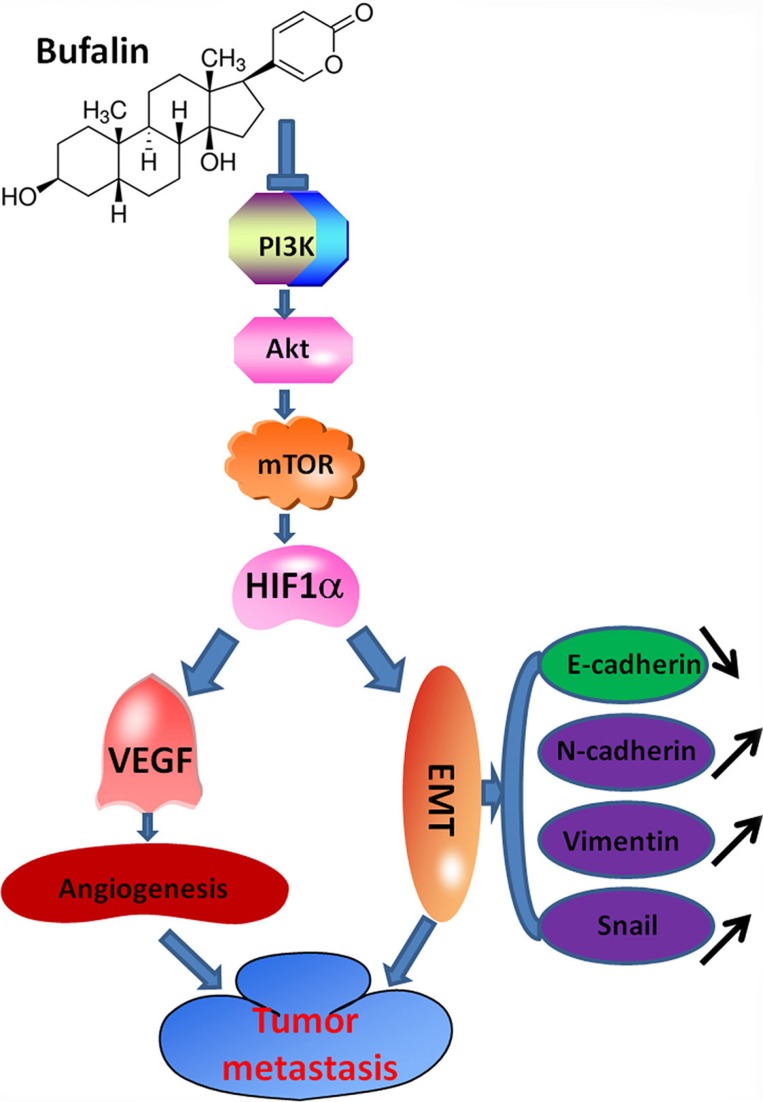
Diagram of the proposed mechanism by which bufalin inhibits hepatocellular carcinoma invasion and metastasis Bufalin inhibited hepatocellular carcinoma invasion and metastasis by inhibiting the EMT and angiogenesis. The antimetastasis effect of bufalin occurs mainly through the downregulation of HIF-1α by the inhibition of the PI3K/Akt/mTOR signaling pathway in HCC. This study indicates that HIF-1α can be a potential anti-cancer target for bufalin.

In summary, we found EMT and angiogenesis played a key role in bufalin inhibited-invasion and metastasis of hepatocellular carcinoma *in vivo* and *in vitro*. Importantly, the downregulation of HIF-1α by the inhibition of the PI3K/Akt/mTOR signaling pathway might regulate entire process. The diagram describes the proposed mechanism (Figure 9).

## DISCUSSION

The incidence of HCC has been increasing in the last two decades and the prognosis remains dismal [1, 3]. Metastasis from the primary tumor to other organs is ubiquitous among HCC patients, which accounts for 90% deaths [25]. Therefore, many efforts have been made to inhibit the metastatic property of HCC. Bufalin, as one of the potent component extracted from Chinese Medicine, has been shown to exert antitumor effect by inhibiting proliferation, angiogenesis and inducing apoptosis [6]. To date, relatively fewer works have been published documenting bufalin's role in metastasis. Recently, the effect of bufalin on metastasis has been given special attention to in different types of cancers. It has been demonstrated that bufalin can suppress the migration and invasion through the inactivation of matrix metalloproteinases and modulation of tight junctions in T24 bladder carcinoma cell lines [7]. Additionally, bufalin can inhibit EMT by downregulating TGF-β receptors in A549 cells [26]. With regard to the role of bufalin in hepatocellular carcinoma, two studies have shown that inhibition of AKT/GSK3β/β-catenin/E-cadherin signaling pathway may be involved in its antimetastatic property [27–28]. In the absence of other known means of bufalin's activity against migration and invasion, we hypothesized that there are other mechanisms accounting for bufalin's antimetastatic property.

In this study, we investigated the role of bufalin on hepatic tumor invasion and metastasis both *in vivo* and *in vitro*. Consistent with previous reports concerning bufalin's role in orthotopic transplantation tumor models injected with HCCLM3-R cells, our results showed that bufalin inhibits HCC metastasis in an orthotopic xenograft model with implanted SMMC7721 cells. Moreover, the antimetastatic activity of bufalin was also confirmed in another tumor metastasis model, which was established by injecting tumor cells via the tail vein.

Furthermore, bufalin significantly downregulated mesenchymal markers and increased the expression of the epithelial markers *in vivo* and *in vitro*, demonstrating that bufalin can inhibit HCC invasion and metastasis by regulating EMT. Thus, we explored the antimetastatic effect of bufalin by analyzing its effects on regulating EMT, which differs from the study performed by Zhang focusing on AKT/GSK3β/β-catenin/E-cadherin pathway [27]. As hepatic tumors have a high tendency to occur as intrahepatic and lung metastases, hepatic tissue and lung tissue were used as parameters to measure hepatic tumor metastasis. Additionally, we established two types of tumor models to observe tumor metastasis. Compared with the control tumors, fewer metastatic lesions were detected in the bufalin-treated mice, especially in the hepatic and lung tissue. Increasing evidence has shown that the EMT plays a key role in tumor metastasis [8]. There is solid evidence indicating that the EMT gives rise to the dissemination of single carcinoma cells from the site of the primary tumor [29]. The inhibition of hepatic carcinoma metastasis by bufalin *in vivo* prompted us to examine whether the antimetastatic effect of bufalin was due to the inhibition of EMT. In agreement with our hypothesis, our *in vivo* results showed bufalin significantly upregulated E-cadherin and downregulated N-cadherin, vimentin, and Snail, which is an important transcriptional repressor of the E-cadherin expression and a regulator of the EMT process in the epithelial cells. However, when we conducted cellular invasion and migration assays, the cell invasion and migration was not significantly reduced in the bufalin-treated group compared with the control group. This phenomenon may be attributed to the low metastatic potential of SMMC7721 cells. To observe the inhibitory effect of bufalin on the invasion and metastasis of the hepatoma cells, we used cytokines to stimulate the hepatoma cells.

TGF-β1, a multifunctional cytokine that regulates cell proliferation, differentiation and apoptosis, is a major inducer of the EMT [30]. TGF-β1 is a known inducer of EMT and is proposed to facilitate cancer progression during late-stage disease [31–33]. Therefore, during the late stages of HCC tumorigenesis, TGF-β1 stimulates cellular invasion through the EMT [34].

In our study, we successfully induced invasion, migration and the EMT, as evidenced by the downregulation of E-cadherin and the upregulation of N-cadherin, vimentin and Snail in TGF-β1-stimulated cells. As expected, these effects were attenuated by bufalin. A study performed by Zhao has demonstrated that bufalin can suppress TGF-β-induced EMT and migration in A549 cells. Their further investigations have revealed that bufalin can inhibit TGF-β receptor I and II, thus attenuating EMT [26].

In human cells, HIF-1α, a signal transcription factor, is widely expressed in a hypoxic environment and plays an important role in tumorigenesis, development, invasion, metastasis and apoptosis [35]. Several studies have demonstrated that HIF-1α may predict tumor diagnosis and recurrence and monitor tumor invasion and metastasis [36–39]. HIF-1α involves two aspects in tumor invasion and metastasis. On the one hand, HIF-1α plays a key role in the VEGF signaling pathway and can increase the expression of VEGF mRNA, which stimulates angiogenesis [22, 40]. Clinical research has reported that HIF-1a and VEGF might be useful as a molecular prediction model for the lymph node metastasis of HCC [22]. On the other hand, HIF-1α may be a master regulator of the EMT [14]. In HCC cells, HIF-1α can promote EMT by increasing Snail transcription [19]. Clinical data have indicated that HIF-1α overexpression is associated with the poor prognosis of HCC, and HIF-1α has been identified as a potential target for HCC therapy [41–42]. Interestingly, studies have shown that HIF-1α is a critical target of cardiac glycosides for cancer therapy [43–44]. Thus, bufalin, a cardiac glycoside drug, should also have an inhibitory effect on HIF-1α.

Based on the role of HIF-1α in modulating EMT in HCC cells and the potential role of bufalin in targeting HIF-1α as a cardiac glycoside, we investigated the role of bufalin in HIF-1α. As expected, there was an evident decrease in HIF-1α expression in SMMC7721 exposed to bufalin treatment.

The signaling mechanisms that lead to HIF-1α upregulation have been a subject of intense investigation. Recent studies proved that PI3K/AKT, RAS/MAPK and NF-κB pathways are involved in the regulation of HIF-1α [23]. In HCC, the PI3K/AKT/mTOR pathway is frequently overactivated, and a clinical study has reported that the activation of the PI3K/ AKT/mTOR pathway correlates with tumor progression and reduced patient survival [45–46]. A recent study has shown that the PI3K/ AKT/mTOR pathway leads to normoxic HIF-1α activation and is considered a potential therapeutic target [47]. Provided that the PI3K/ AKT/mTOR pathway may regulate HIF-1α expression, we then hypothesized that the PI3K/AKT/mTOR pathway may be involved in bufalin's inhibition of EMT via targeting HIF-1α. As shown in the results, we observed that bufalin inhibited HIF-1α expression, which was in accordance with the inhibition of p-AKT and mTOR expressions. As we have shown in our present study, there were incomplete inhibitions of HIF-1α expression by PI103, the PI3K inhibitor and MK2206, the AKT inhibitor, respectively. Besides, HIF-1α expreesion was partially abolished by siRNA targeting AKT. The following explanations maybe possible: there are many upstream pathways of HIF-1α rather than PI3K/AKT/mTOR alone, thus the suppression of one mere pathway would not be sufficient to abolish HIF-1α expression. One pathway may not be completely blocked due to the cross talk with other pathways.

Interestingly, the RAS/MAPK pathway is activated in this process. However, there is a recent paper that proposed an alternative mechanism of bufalin-mediated reduction of metastasis, notably through inactivation of ERK1/2 and NF-kB signalling pathways. We assumed that the discrepancy between Chen's and our study can be attributed to the following: Applications of different of HCC cell lines may lead to the discrepancies in the result. 2) In our study, the RAS/MAPK pathway is activated in this process. Previous data and computer simulations demonstrate that there is cross talk between the PI3K/Akt pathway and the RAS/MAPK pathway that can activate or inhibit each other [48]. In HCC, the cross talk between the PI3K/Akt pathway and the MEK/ERK pathway cascade is a protective mechanism to adapt to endoplasmic reticulum (ER) stress [24]. To further validate the potential role of HIF-1α in HCC cells exposed to bufalin in EMT, we detected bufalin's effect on EMT in SMMC7721 cells combined with employment of HIF-1α siRNA. Not surprisingly, downregulation of HIF-1α reversed EMT, and the inhibition of EMT augmented in SMMC7721 cells when combined with bufalin, as evidenced by the significant upregulation of E-cadherin, downregulation of N-cadherin, Snail, Vimentin.

As shown in Figure 6F and 6G, the MVD and VEGF expressions in the lungs were downregulated in bufalin-treated mice. In recent studies, researchers have found that PI3K/AKT/mTOR and HIF-1α pathway has been involved in the VEGF-A-dependent angiogenesis in human chondrosarcoma induced by adiponectin [54]. However, whether bufalin-induced VEGF reduction was linked with PI3K/AKT/mTOR/ HIF-1α pathway needs to be further tested, possibly by the expressions of VEGF using HIF-1α siRNA and PI3K, AKT inhibitors.

The confirmed direct targets of bufalin include fibronectin, TGFβ receptor I and II and SRC, as some studies have reported. And in our present study, we have identified HIF-1α as one of the direct targets of bufalin. Other indirect targets of bufalin include important molecules in the pathways which bufalin modulates. For instance, E-cadherin, Wnt and β-catenin, MMPs, mitogen-activated protein kinases (MAPKs) and NF-κB systems are the indirect targets that bufalin modulated. In conclusion, bufalin, an effective component of cinobufacini, extracted from toad cake, is an antimetastatic drug of multi-targets, both in direct and indirect means, which justifies the multi-targets of Chinese medicine.

In conclusion, our study shows that bufalin suppresses hepatic tumor metastasis by targeting HIF-1α through the inhibition of the PI3K/AKT/mTOR pathway. Therefore, this study provides theoretical support for cinobufacini's antimetastatic effect. And further studies can be performed in HCC patients to warrant its clinical relevance.

## MATERIALS AND METHODS

### Reagents and antibodies

Bufalin and dimethyl sulfoxide (DMSO) were obtained from Sigma Chemical Co (St. Louis, MO, USA). Antibodies against E-cadherin, N-cadherin, vimentin, HIF-1α, and CD31 were purchased from Abcam (Cambridge, UK); anti-Snail and anti-VEGF were purchased from Bioworld (Bioworld Technology, Minneapolis, MN, USA); and recombinant transforming growth factor-β1 (TGF-β1) was purchased from R & D Systems (Minneapolis, MN, USA); The specific primary antibodies for mTOR, phospho-mTOR, AKT, phospho-AKT, PI3K, phospho-PI3K, ERK1/2, phospho-ERK1/2, JNK, phospho-JNK, p38 MAPK, phospho-p38 MAPK, and GAPDH were purchased from Cell Signaling Technology (Danvers, MA, USA); The PI3K/AKT inhibitors including PI103 and MK-2206 were purchased from Selleck Chemicals LLC (Houston, TX, USA). The siRNA-AKT and siRNA-mTOR were purchased from Cell Signaling Technology (Danvers, MA, USA).

### Cell culture

The human HCC cell line SMMC7721 was obtained from the American Type Culture Collection and was cultured in RPMI-1640 medium containing 10% fetal bovine serum (FBS; Gibco, Carlsbad, CA, USA) in 5% CO_2_ at 37°C. The SMMC7721 cells that were transfected with the green fluorescence protein (GFP) were labeled SMMC7721-GFP cells.

### Cell transfection with green fluorescence protein (GFP)

Human HCC cells SMMC7721 were maintained in RPMI 1640 with 10% FBS. 293T cells were grown in DMEM containing 10% FBS. Retroviral vector, MSCV-GFP, encoding a retroviral packaging plasmid was employed. To generate pseudotyped virus, MSCV-GFP vectors were into subconfluent 293T cells. The virus stocks were collected at 48 and 72 h after transfection, filtered through a 0.45 μm filter, and then frozen at −80°C. Human HCC SMMC7721 cells were infected with retrovirus stocks containing 8 μg/ml polybrene for 6 h, washed, and cultured in fresh complete medium. Repeated infections were conducted on subsequent days, after an identical procedure.

### Animal models and treatments

Six-week-old BALBc nu/nu mice were obtained from the Shanghai Institute of Material Medica, Chinese Academy of Science. The mice were bred in laminar flow cabinets under pathogen-free conditions. We followed internationally recognized guidelines on animal welfare. The study design was approved by the Animal Ethics Committee, and the experiments were undertaken in accordance with the ethical principles of the Animal Experimentation of Fudan University.

The SMMC7721-GFP cells (5 × 10^6^) were subcutaneously inoculated into the right flanks of 6-week-old BALBc nu/nu mice. After four weeks, the non-necrotic tumor tissue was cut into 1-mm^3^ pieces and orthotopically implanted into the liver. The treatment was initiated one week later. The mice were randomly separated into two groups with six mice per group. The mice in the experimental group received intraperitoneal injections of 1 mg/kg bufalin (5 days/week), whereas the control mice were injected with the vehicle alone (PBS). The treatment was continued for six weeks; then, the mice were sacrificed, and the tumors were excised from each mouse, weighed and snap-frozen for further analysis.

In addition, SMMC7721-GFP cells (2 × 10^6^/0.2 ml of phosphate-buffered saline, PBS) were injected via mouse tail veins. The treatment was initiated one week later. The mice were randomly divided into two groups (*n* = 9 per group). The mice in the experimental group received intraperitoneal injections of 1 mg/kg bufalin (5 days/week), whereas the control mice were injected with vehicle alone. The mouse weights were measured twice a week for 8 weeks, and then the mice were sacrificed. The lungs were excised from each mouse for further analysis.

### Detection of metastasis

In the orthotopic xenograft model, metastasis was evaluated using the Inveon micro PET/CT system (Siemens, Knoxville, TN, USA). The liver was excised, and intrahepatic metastasis was evaluated using the Lumazone imaging system (Mag Biosystems, Tucson, AZ, USA). The green fluorescent protein-positive metastatic foci were imaged. Then, the tumors were excised, and their largest (a) and smallest (b) diameters were measured to calculate the tumor volume (V = ab^2^/2). The lungs were also excised, and HE staining was applied to detect lung and liver metastases. In the tail vein metastasis model, the systemic metastases were also quantified using the Lumazone imaging system. In addition, HE staining was applied to detect lung metastasis.

### Cell proliferation, migration and invasion assays

The cell proliferation analysis was performed as described previously [20]. Briefly, cells were plated at 5000 cells per well in 96-well microtiter plates and incubated overnight at 37°C in a humidified incubator containing 5% CO_2_. The following day, various concentrations of bufalin were added to the wells, and the cultures were incubated for an additional 24, 48, or 72 h. Cell viability was determined using a Cell Counting Kit-8 (Dojindo, Gaithersburg, MD, USA) according to the manufacturer's instructions. For the cell migration assay, the cell migration was assessed using the transwell assay (Boyden Chambers, Corning, and Cambridge, MA, USA).

Cells (5 × 10^4^) were seeded in a serum-free medium in the upper chamber and allowed to migrate towards the lower chamber that contained 10% FBS. After 48 h, a cotton swab moistened with medium was inserted into the chamber to get rid of the cells on the upper surface of the membrane. The cells were fixed using 4% paraformldehyde and stained with 0.1% crystal violet. The migrated cells were counted in five selected fields under a microscope at a 20× magnification. The cell invasion assay was performed similarly, except that 50 μl of Matrigel (BD Biosciences, Franklin Lakes, NJ, USA) that was diluted 1:6 with serum-free medium, was added to each well overnight before the cells (2 × 10^5^) were seeded onto the membrane.

### TGF-β1 treatment

The cells were plated in a 10-cm cell culture dish at a density of 1 × 10^6^ cells/well and left overnight. The next day, the cells were serum-starved overnight, and the media were replaced with fresh media containing 10 ng/ml TGF-β1 and 2% FBS. In the bufalin-treated group, 5 nM bufalin was added to the TGF-β1-treated cells. After 72 h of incubation, the cells were collected for further analysis.

### Western blot analysis

The cells were washed with cold PBS and lysed in the culture dishes using a PhosphoSafe^™^ Extraction Reagent (Merck, Darmstadt, Germany) containing 1% Protease Inhibitor Cocktail (EDTA-free, Thermo, San Jose, CA, USA). The protein concentrations were then determined using the Bio-Rad detergent compatible protein assays (Bio-Rad, Hercules, CA, USA). Protein from the control and treated cell lysates was loaded onto 8% to 12% gradient NuPAGE gels (Novex, San Diego, CA, USA), electrophoresed under reducing conditions, and transferred onto polyvinylidene difluoride membranes (0.22 μm; Millipore). Western blot analysis was performed as described previously [20].

### Transfection of HIF-1α siRNA

SMMC7721 cells were plated in 6-well plates and allowed to attach overnight. Transient transfection of scramble siRNA and HIF-1α siRNA was carried out using Neofect Transfection Reagent following the instructions of the manufacturer

### Immunohistochemistry

Immunohistochemical analysis was performed as described previously [21]. Briefly, the tumor sections were stained with rabbit anti-E-cadherin, rabbit anti-N-cadherin, rabbit anti-vimentin, rabbit anti-HIF-1α, rabbit anti-CD31, rabbit anti-VEGF, and mouse anti-Snail at 4°C overnight. The goat anti-rabbit or mouse IgG/horseradish peroxidase was applied as the secondary antibody according to the standard protocols provided by the manufacturer. For negative controls, the primary antibodies were replaced with PBS.

Immunopositivity was determined by two independent investigators, both of whom were blinded to the model/treatment type for the series of experiments. Protein expression was evaluated according to the following formula: overall score = intensity score × percentage score. The intensity was graded as follows: 0, negative; 1, weak; and 2, strong. The ratio of the positive cells per specimen was evaluated quantitatively and scored as follows: 0, ≤ 10%; 1, 11 to 25%; 2, 26 to 50%; 3, 51 to 75%; 4, > 75%. Thus, a total score of 0 to 8 was calculated.

### Immunofluorescence

The cells were fixed with 4% paraformaldehyde. After washing with PBS, the cells were blocked with 5% bovine serum albumin (BSA) and incubated with anti-vimentin (1:100) and anti-E-cadherin (1:100) at 4°C overnight. The cells were subsequently incubated with a fluorescence-conjugated secondary antibody for 1 h. Then, the cells were mounted with a mounting medium containing DAPI (Vector Laboratories). For the negative controls, the primary antibodies were replaced with PBS. The images were captured using a confocal Leica fluorescence microscope.

### Real-time polymerase chain reaction (RT-PCR)

Real-time polymerase chain reaction (RT-PCR) analysis was performed as described previously [22]. The following primers for the amplification of the human genes were used: E-cadherin, forward 5′- AGCCCCGCCTTATGATTCTCTG-3′ and reverse 5′-TGCCCCATTCGTTCAAGTAGTCAT-3′; N-cadherin, forward 5′-CCACGCCGAGCCCCAGTAT-3′ and reverse 5′-GGCCCCCAGTCGTTCAGGTAAT-3′; vimentin, forward 5′-CCTTGACATTGAGATTGCCACCTA-3′ and reverse 5′-TCATCGTGATGCTGAGAAGTTTCG-3′; and Snail, forward 5′-CAGCCTGGGTGCCCTCAAGAT-3′ and reverse 5′-GCACACGCCTGGCACTGGTA-3′. GAPDH, forward 5′-ATGAATTCAGGTGAAGGTCG GAGTCAACG -3′, and reverse 5′-ATGGATCCAGGCT GTTGTCATACTTCTC-3′, which were used for cDNA amplification. PCR conditions consisted of 40 amplification cycles, each at 95°C for 15 s, 57°C for 30 s, and 72°C for 30 s. E-cadherin, N-cadherin, Vimentin, Snail genes were normalized against housekeeping GAPDH gene using the 2−ΔΔCt method.

### Enzyme-linked immunosorbent (ELISA) assay

The whole-blood samples of the mice were mixed with heparin, centrifuged (12000 × g) for 15 min, and then cryopreserved at −80°C. Serum VEGF levels were measured using a sandwich ELISA kit (R & D Systems, Minneapolis, MN, USA) according to the manufacturer's instructions and analyzed using a Labsystems Multiscan reader (Thermo Fisher Scientific, MA, USA).

### Statistical analyses

The results were analyzed using the GraphPad prism version 5.0 (GraphPad Software, San Diego, CA, USA) and the SPSS 19.0 software package (SPSS Inc, Chicago, IL, USA). The statistical analyses were performed using the chi-squared tests, Student's *t*-test and the analysis of variance (ANOVA) models. The level of significance was set at *P*< 0.05.

## References

[1] Jemal A, Bray F, Center MM, Ferlay J, Ward E, Forman D (2011). Global cancer statistics. CA Cancer J Clin.

[2] Gao JJ, Song PP, Tamura S, Hasegawa K, Sugawara Y, Kokudo N, Uchida K, Orii R, Qi FH, Dong JH, Tang W (2012). Standardization of perioperative management on hepato-biliary-pancreatic surgery. Drug Discov Ther.

[3] Belghiti J, Fuks D (2012). Liver resection and transplantation in hepatocellular carcinoma. Liver Cancer.

[4] Cheah YL, Chow P (2012). Liver transplantation for hepatocellular carcinoma: an appraisal of current controversies. Liver Cancer.

[5] Schwartz M, Roayaie S, Llovet J (2005). How should patients with hepatocellular carcinoma recurrence after liver transplantation be treated?. J Hepatol.

[6] Qi F, Li A, Inagaki Y, Kokudo N, Tamura S, Nakata M, Tang W (2011). Antitumor activity of extracts and compounds from the skin of the toad Bufo bufo gargarizans Cantor. Int Immunopharmacol.

[7] Hong SH, Kim GY, Chang YC, Moon SK, Kim WJ, Choi YH (2013). Bufalin prevents the migration and invasion of T24 bladder carcinoma cells through the inactivation of matrix metalloproteinases and modulation of tight junctions. Int J Oncol.

[8] Lee JM, Dedhar S, Kalluri R, Thompson EW (2006). The epithelial-mesenchymal transition: new insights in signaling, development, and disease. J Cell Biol.

[9] Wang Y, Wen M, Kwon Y, Xu Y, Liu Y, Zhang P, He X, Wang Q, Huang Y, Jen KY, LaBarge MA, You L, Kogan SC (2014). CUL4A induces epithelial-mesenchymal transition and promotes cancer metastasis by regulating ZEB1 expression. Cancer Res.

[10] Franco-Chuaire ML, Magda Carolina SC, Chuaire-Noack L (2013). Epithelial-mesenchymal transition (EMT): principles and clinical impact in cancer therapy. Invest Clin.

[11] Yang MH, Chen CL, Chau GY, Chiou SH, Su CW, Chou TY, Peng WL, Wu JC (2009). Comprehensive analysis of the independent effect of twist and snail in promoting metastasis of hepatocellular carcinoma. Hepatology.

[12] Zhou Z, Zhao C, Wang L, Cao X, Li J, Huang R, Lao Q, Yu H, Li Y, Du H, Qu L, Shou C (2015). A VEGFR1 antagonistic peptide inhibits tumor growth and metastasis through VEGFR1-PI3K-AKT signaling pathway inhibition. Am J Cancer Res.

[13] Myung SJ, Yoon JH (2007). Hypoxia in hepatocellular carcinoma. The Korean journal of hepatology.

[14] Luo D, Wang Z, Wu J, Jiang C, Wu J (2014). The role of hypoxia inducible factor-1 in hepatocellular carcinoma. Biomed Res Int.

[15] Xiao H, Tong R, Ding C, Lv Z, Du C, Peng C, Cheng S, Xie H, Zhou L, Wu J, Zheng S (2015). Y-H2AX promotes hepatocellular carcinoma angiogenesis via EGFR/HIF-1α/VEGF pathways under hypoxic condition. Oncotarget.

[16] Rigiracciolo DC, Scarpelli A, Lappano R, Pisano A, Santolla MF, De Marco P, Cirillo F, Cappello AR, Dolce V, Belfiore A, Maggiolini M, De Francesco EM (2015). Copper activates HIF-1α/GPER/VEGF signalling in cancer cells. Oncotarget.

[17] Krishnamachary B, Zagzag D, Nagasawa H, Rainey K, Okuyama H, Baek JH, Semenza GL (2006). Hypoxiainducible factor-1-dependent repression of E-cadherin in von Hippel-Lindau tumor suppressor-null renal cell carcinoma mediated by TCF3, ZFHX1A, and ZFHX1B. Cancer Research.

[18] Yang MH, Wu MZ, Chiou SH, Chen PM, Chang SY, Liu CJ, Teng SC, Wu KJ (2008). Direct regulation of TWIST by HIF-1alpha promotes metastasis. Nature Cell Biology.

[19] Zhang L, Huang G, Li X, Zhang Y, Jiang Y, Shen J, Liu J, Wang Q, Zhu J, Feng X, Dong J, Qian C (2013). Hypoxia induces epithelial mesenchymal transition via activation of SNAI1 by hypoxia inducible factor-1α in hepatocellular carcinoma. BMC Cancer.

[20] Liu Y, Zhang JB, Qin Y, Wang W, Wei L, Teng Y, Guo L, Zhang B, Lin Z, Liu J, Ren ZG, Ye QH, Xie Y (2013). PROX1 promotes hepatocellular carcinoma metastasis by way of up-regulating hypoxia inducible factor 1α expression and protein stability. Hepatology.

[21] Guo R, Meng Q, Guo H, Xiao L, Yang X, Cui Y, Huang Y (2016). TGFβ-2 induces epithelial-mesenchymal transition in cultured human lens epithelial cells through activation of thePI3K/Akt/mTOR signaling pathway. Mol Med Rep.

[22] Xiang ZL, Zeng ZC, Fan J, Tang ZY, Zeng HY, Gao DM (2011). Gene expression profiling of fixed tissues identified hypoxia-inducible factor-1α, VEGF, and matrix metalloproteinase-2 as biomarkers of lymph node metastasis in hepatocellular carcinoma. Clin Cancer Res.

[23] Jiang H, Zhu YS, Xu H, Sun Y, Li QF (2010). Inflammatory stimulation and hypoxia cooperatively activate HIF-1{alpha} in bronchial epithelial cells: involvement of PI3K and NF-{kappa}B. Am J Physiol Lung Cell Mol Physiol.

[24] Dai R, Chen R, Li H (2009). Cross-talk between PI3K/Akt and MEK/ERK pathways mediates endoplasmic reticulum stress-induced cell cycle progression and cell death in human hepatocellular carcinoma cells. Int J Oncol.

[25] Qiao M, Sheng S, Pardee AB (2008). Metastasis and AKT activation. Cell Cycle.

[26] Zhao L, Liu S, Che X, Hou K, Ma Y, Li C, Wen T, Fan Y, Hu X, Liu Y, Qu X (2015). Bufalin inhibits TGFβ-induced epithelial-to-mesenchymal transition and migration in human lung cancer A549 cells by downregulating TGFβ- receptors. Int J Mol Med.

[27] Zhang ZJ, Yang YK, Wu WZ (2014). Bufalin attenuates the stage and metastatic potential of hepatocellular carcinoma in nude mice. J Transl Med.

[28] Qiu DZ, Zhang ZJ, Wu WZ, Yang YK (2013). Bufalin, a component in Chansu, inhibits proliferation and invasion of hepatocellular carcinoma cells. BMC Complement Altern Med.

[29] Thiery JP (2002). Epithelial-mesenchymal transitions in tumor progression. Nat Rev Cancer.

[30] Amin R, Mishra L (2008). Liver stem cells and tgf-Beta in hepatic carcinogenesis. Gastrointest Cancer Res.

[31] Roberts AB, Wakefield LM (2003). The two faces of transforming growth factor beta in carcinogenesis. Proc Natl Acad Sci USA.

[32] Abou-Shady M, Baer HU, Friess H, Berberat P, Zimmermann A, Graber H, Gold LI, Korc M, Büchler MW (1999). Transforming growth factor betas and their signaling receptors in human hepatocellular carcinoma. Am J Surg.

[33] Mishra L, Shetty K, Tang Y, Stuart A, Byers SW (2005). The role of TGF-beta and Wnt signaling in gastrointestinal stem cells and cancer. Oncogene.

[34] Xu J, Lamouille S, Derynck R (2009). TGF beta-induced epithelial to mesenchymal transition. Cell Res.

[35] Semenza GL (2003). Targeting HIF-1 for cancer therapy. Nat Rev Cancer.

[36] Hao J, Song X, Song B, Liu Y, Wei L, Wang X, Yu J (2008). Effects of lentivirus-mediated HIF-1alpha knockdown on hypoxia-related cisplatin resistance and their dependence on p53 status in fibrosarcoma cells. Cancer Gene Ther.

[37] Samulitis BK, Landowski TH, Dorr RT (2009). Inhibition of protein synthesis by imexon reduces HIF-1alpha expression in normoxic and hypoxic pancreatic cancer cells. Invest New Drugs.

[38] Burrows N, Resch J, Cowen RL, von Wasielewski R, Hoang-Vu C, West CM, Williams KJ, Brabant G (2010). Expression of hypoxia-inducible factor 1 alpha in thyroid carcinomas. Endocr Relat Cancer.

[39] Seeber LM, Horrée N, van der Groep P, van der Wall E, Verheijen RH, van Diest PJ (2010). Necrosis related HIF-1alpha expression predicts prognosis in patients with endometrioid endometrial carcinoma. BMC Cancer.

[40] Vumbaca F, Phoenix KN, Rodriguez-Pinto D, Han DK, Claffey KP (2008). Double-stranded RNA-binding protein regulates vascular endothelial growth factor mRNA stability, translation, and breast cancer angiogenesis. Mol Cell Biol.

[41] Zheng SS, Chen XH, Yin X, Zhang BH (2013). Prognostic significance of HIF-1α expression in hepatocellular carcinoma: a meta analysis. PLoS ONE.

[42] Cao S, Yang S, Wu C, Wang Y, Jiang J, Lu Z (2014). Protein expression of hypoxia inducible factor-1 alpha and hepatocellular carcinoma: a systematic review with meta-analysis. Clinics and Research in Hepatology and Gastroenterology.

[43] Lin J, Carducci MA (2009). HIF-1alpha inhibition as a novel mechanism of cardiac glycosides in cancer therapeutics. Expert Opin Investig Drugs.

[44] Zhang H, Qian DZ, Tan YS, Lee K, Gao P, Ren YR, Rey S, Hammers H, Chang D, Pili R, Dang CV, Liu JO, Semenza GL (2008). Digoxin and other cardiac glycosides inhibit HIF-1alpha synthesis and block tumor growth. Proc Natl Acad Sci USA.

[45] Tommasi S, Pinto R, Pilato B, Paradiso A (2007). Molecular pathways and related target therapies in liver carcinoma. Curr Pharm Des.

[46] Sun CH, Chang YH, Pan CC (2011). Activation of the PI3K/Akt/mTOR pathway correlates with tumour progression and reduced survival in patients with urothelial carcinoma of the urinary bladder. Histopathology.

[47] Agani F, Jiang BH (2013). Oxygen-independent regulation of HIF-1: novel involvement of PI3K/AKT/mTOR pathway in cancer. Curr Cancer Drug Targets.

[48] Aksamitiene E, Kiyatkin A, Kholodenko BN (2012). Cross-talk between mitogenic Ras/MAPK and survival PI3K/Akt pathways: a fine balance. Biochem Soc Trans.

[49] Wang Y, Lonard DM, Yu Y, Chow DC, Palzkill TG, Wang J, Qi R, Matzuk AJ, Song X, Madoux F, Hodder P, Chase P, Griffin PR (2014). Bufalin is a potent small-molecule inhibitor of the steroid receptor coactivators SRC-3 and SRC-1. Cancer Res.

